# Fractal-based compact quad-port THz MIMO antenna with ultra-wideband and high isolation for 6G and TWPAN applications

**DOI:** 10.1038/s41598-025-19595-2

**Published:** 2025-10-13

**Authors:** Annadurai Chinnamuthu, Nelson Iruthayanathan, S. Ramprabhu, R. Vijaya Arjunan, Tanuja Shailesh

**Affiliations:** 1 Department of ECE, Sri Sivasubramaniya Nadar College of Engineering, Chennai, 603 110 India; 2 Department of Electronics Engineering, Madras Institute of Technology, Anna University, Chennai, 600 044 India; 3 Manipal Institute of Technology, Manipal Academy of Higher Education, Manipal, India

**Keywords:** MIMO, Fractal, THz, Bandwidth, Gain, ECC, TARC, MEG, DG, CCL, Engineering, Physics

## Abstract

This study describes a high-gain, ultra-wideband quad-port THz MIMO antenna designed for 6G, TWPAN, and next-generation wireless communications. The design uses fractal radiating elements, graphene-based tunability, and a defective ground structure (DGS) to improve bandwidth, isolation, and impedance matching. The antenna’s tiny polyimide substrate (130 × 130 μm²) enables an ultra-wide bandwidth of 62 THz, a peak gain of 15.24 dB, and port-to-port isolation of 43 dB, resulting in strong MIMO performance. Integrating metasurface structures and graphene tuning allows for dynamic frequency reconfiguration, making it suitable for a wide range of wireless applications. The performance study reveals good spatial diversity and low signal distortion, with an envelope correlation coefficient (ECC) of less than 0.05 and a Diversity Gain (DG) of nearly 10 dB. Additionally, the Total Active Reflection Coefficient (TARC) and Channel Capacity Loss (CCL) are kept to a minimum, maximising spectral efficiency. UV photolithography and electron beam evaporation (EBE) are employed to fabricate the antenna, yielding high precision and minimal losses. Compared to existing designs, it outperforms them in terms of gain, isolation, and multi-band operation. The suggested THz MIMO antenna’s scalability, compact form factor, and customisable properties make it an attractive choice for future 6G wireless networks, sub-THz IoT systems, and ultra-fast personal area networks (PANs). Future research will focus on adaptive beamforming, real-world prototypes, and experimental validation to improve its application in next-generation THz communication systems.

## Introduction

Over the past decade, wireless communication has undergone significant evolution from low-speed 2G/3G networks to high-speed 5G/6G technologies. The demand for higher speed and bandwidth has surged substantially. Researchers are working on 5G and 6G networks to meet this growing demand. Most countries have deployed 5G networks, offering high-speed connectivity with significant data rates and enabling seamless streaming of premium content. Scientists are now focused on further advancements by developing 6G networks, which generally operate in the high GHz or THz frequency range. The development of 6G networks requires high-gain, wideband THz antennas capable of supporting high-speed data transmission over wider bandwidths^1^.

High-speed MIMO antenna designs play a crucial role in meeting the demands of 6G connectivity. With wide bandwidth and high gain, MIMO antennas are practical for 5G communication systems. MIMO antennas incorporating metamaterial elements demonstrate superior performance compared to conventional designs. Two-port MIMO antennas with split-ring resonator metamaterial loading enhance design characteristics, including scattering parameters, gain, and bandwidth. One such MIMO design has shown excellent results with a split-ring resonator. Metamaterial-based MIMO antennas enable an ultrawideband response. Double-negative metamaterial loading, essential for compact and portable devices, can also help reduce antenna size. Both metamaterials and graphene can be integrated to make THz MIMO antennas suitable for high-speed applications like 6G^2^. Table 1 provides an overview of the diverse applications of the THz spectrum across different frequency ranges.

**Table 1:** Detailed table outlining the applications of the Terahertz (THz) spectrum from 0.1 to 100 THz with relevant key applications.

Refs.	Frequency band	Range (THz)	Key applications
^3^	Sub-THz communications	0.1–0.3	6G wireless networks, short-range high-data-rate communication, automotive radar
^4^	THz imaging & security	0.3–1.0	Airport security scanners, non-destructive testing (NDT), concealed object detection
^5^	Biomedical & spectroscopy	1.0–3.0	Skin and tissue imaging, cancer detection, thz spectroscopy for biomolecules
^6^	High-speed THz communications	3.0–10	Ultra-fast wireless data transfer, optical fibre integration, inter-satellite links
^7^	Quantum & material research	10–30	Semiconductor physics, graphene-based devices, THz waveguides for quantum computing
^8^	Chemical & molecular sensing	30–60	Gas and pollutant detection, atmospheric sensing, remote chemical explosive detection
^9^	Ultra-fast electronics & optoelectronics	60–100	Next-gen photonic circuits, high-speed processors, THz-driven lasers

Reference^10^ offers a 4-port MIMO antenna with compact high gain. A three-phase analysis determines the ideal form of the radiating element. Design optimisation involves adjusting the ground layer length and the diameter of the circular patch. Reversing loss values of -10.27 dB, -18.51 dB, and − 18.59 dB, the final construction measures 78 × 50 mm². With a gain of 10.03 dBi, the suggested device produces a peak bandwidth of 3.94 GHz. We investigate closely key MIMO performance measures like TARC, ECC, MEG, DG, and CCL. Simulated and measured findings validate the efficacy of the design, making it suitable for 5G, Wi-Fi, and WLAN applications. For antenna systems, radomes serve as protective casings, shielding them from hostile environments. Still, they can compromise the antenna’s electromagnetic performance^11^. Under independently and identically distributed, flat Rayleigh fading circumstances, the ergodic channel capacities of several wireless communication systems are investigated and compared in another work^12^. Simulated and theoretical experiments demonstrate that the ergodic capacity of MIMO channels increases linearly with the number of antennas. Substantial MIMO performance criteria have been effectively designed in a well-optimised MIMO antenna^13^. The design also includes circular polarisation, qualifying for THz spectrum operation. Graphene improves its filtering properties, increasing the range of applications^14^. The MIMO antenna can achieve a multiband response within the THz frequency range by integrating metamaterials. One realises this effect with graphene, a material with excellent tuning capacity^15^. Combining graphene with a metasurface develops a reasonably affordable broadband antenna design. By altering the potential of graphene, one can modify the antenna’s frequency response, making it suitable for various wireless applications^16^. Furthermore, improving the versatility of the antenna for multiple uses, such as WiMAX^17^, involves defective ground structures. THz MIMO antennas, including defective ground structures, have been investigated for high-speed wireless communication in 5G, 6G, and beyond^17,18^. Furthermore, sub-THz spectrum operation of antennas meant for 6G and IoT is robust. We have simulated these antennas using HFSS and CST Microwave Studio software^22^.

Article^19^ The hybrid metasurface sensors based on advanced 2D materials have shown remarkable potential in early-stage brain tumour diagnostics. By combining high-Q resonant terahertz structures with machine learning classification, these systems can identify subtle dielectric changes in tissues, offering higher precision than conventional imaging modalities. Metamaterial-clad terahertz fibres incorporating superconducting layers enable ultra-low-loss and dispersion-controlled signal transport. Such guiding mechanisms ensure strong mode confinement and minimal attenuation, making them valuable for high-performance THz MIMO communication links^20^. Ultra-wideband cross-polarisation converters in the THz range can be engineered to also serve as sensitive biosensors. The metasurface configuration ensures efficient polarisation transformation while enabling precise detection of molecular spectral signatures^21^.

Hybrid metasurfaces that combine multiple resonator types offer enhanced spectral selectivity for the detection of tuberculosis biomarkers. Machine learning models further refine detection accuracy, allowing rapid and reliable diagnosis at terahertz frequencies^22^.

Article^23^ Demonstrates that MXene–gold plasmonic architectures in the THz band exhibit substantial resonant field enhancement, enabling the sensitive detection of bacterial biomarkers. Machine learning aids in spectral interpretation, reducing noise and improving diagnostic reliability. Graphene–gold hybrid metasurfaces offer tunable plasmonic responses for detecting Isoquercitrin with high precision. Coupled with machine learning analysis, these sensors achieve superior sensitivity and robust noise suppression in THz detection systems^24^. Multilayer metamaterial absorbers have been optimised for broadband solar–thermal conversion using random forest regression models. Such predictive design strategies accelerate material selection for integrated THz energy-harvesting applications^25^. Reflective metasurface-based converters in the THz domain can maintain polarisation conversion efficiency over wide bandwidths and angles. Incorporating biosensing functionality allows simultaneous communication and molecular detection^26^.

Two-dimensional material metasurfaces enable tunable, high-sensitivity detection in the terahertz range through electrical biasing. Machine learning validation ensures accurate signal interpretation, enhancing real-time sensing performance^27^. The Circular SPR biosensors with graphene–MXene–Au layers demonstrate exceptional glucose detection capability. AI-driven analysis refines sensitivity, making it suitable for integration into wireless THz health-monitoring systems^28^. Antennas that provide either high gain or wide bandwidth, specially tailored for 5G and 6G uses. The suggested THz MIMO antenna offers outstanding gain and ultra-wide bandwidth. Furthermore, the investigation focuses on MIMO construction using affordable materials to ensure compatibility with low-cost devices. Its small scale helps to improve its fit for portable uses even more.

### Objective of work

This study aims to design and construct a high-gain, ultra-wideband THz MIMO antenna that seamlessly incorporates fractal geometry, graphene-based tuning, and metasurface technology to revolutionise next-generation 6G and TWPAN wireless communications. The suggested antenna architecture centres on the following points:

- Maximising spectrum efficiency with a quad-port MIMO setup that optimises isolation and minimises mutual coupling.
- Achieving ultra-compact scalability to support tiny, high-speed IoT and smart device networks.
- Using a defective ground structure (DGS) and fractal-inspired radiators, we overcome traditional bandwidth limits and achieve robust multi-band operation.

The suggested architecture sets a new bar in 6G wireless infrastructure. It offers exceptional signal integrity, faster data throughput, and energy-efficient transmission, making it perfect for prospective high-speed communication networks.

### Novelty of proposed work

- The proposed THz MIMO antenna employs a fractal geometry that enhances multi-band operation, improves impedance matching, and supports a wideband response.
- The antenna incorporates graphene with metasurface technology, allowing frequency tuning, improved radiation efficiency, and a cost-effective broadband design.
- The design features a DGS, significantly reducing mutual coupling, enhancing isolation and overall MIMO performance.
- Optimisation of design achieved by changing slots’ physical size and the width of the ground layer.
- The proposed antenna achieves a peak gain of 15.24 dB with an ultra-wideband bandwidth of 62 THz, outperforming conventional MIMO designs.
- The structure is optimised to 130 × 130 μm², making it highly suitable for THz-operated 6G, IoT, and high-speed personal area network applications.
- The antenna is designed with quad-port operation, improving spatial diversity and maximising channel capacity while ensuring minimal envelope correlation.

### The flow of work

Section 1 highlights the need for a MIMO antenna and the necessary lecture review, including various band applications in the current era. Section 2 discusses the structure dimensions and the optimisation process. Section 3 discusses different results. The section also presents a comparative analysis of the proposed work with other structures and concluding remarks.

### Design and modelling

Designed terahertz (THz) MIMO antennas now primarily utilise fractal geometry because they can accommodate multiple resonances while maintaining a compact form factor. The effective electrical length of such an antenna determines its resonance frequency, which varies with the number of iterations in the fractal. The resonance frequency is governed fundamentally by Eq. (1)^29^.

1 $$\:{f}_{n}=\frac{c}{2{L}_{n}\sqrt{{\epsilon\:}_{eff}}}$$

2 $$\:{L}_{n}={L}_{0}\times\:{r}^{n}$$

$\:{L}_{n}$​ It is the effective length of the radiating structure, and it is calculated using Eq. (2). $\:c$ Is the speed of light; fn​ is the resonant frequency at the n^th^ iteration; $\:{\epsilon\:}_{eff}$​ Indicates the effective permittivity of the substrate material. Changing the fractal structure makes these highly efficient antennas for THz applications feasible, controlling the resonant frequency and impedance characteristics.

L_0_ is the basis shape’s starting length; r is its fractal scaling factor; n is the iteration level. Each iteration’s antenna construction becomes increasingly complex, allowing it to resonate at multiple frequencies. This quality benefits THz communications, where size restrictions provide significant difficulties. Furthermore, fractal antennas naturally support wideband features and are suitable for next-generation wireless networks, such as 6G and ultra-fast personal area networks (WPANs).

Mutual coupling, the phenomenon in which electromagnetic waves from one antenna element interact with those from another, is one of the primary determinants of MIMO antenna performance. The optimal distance between the antenna elements will help decrease coupling and preserve diversity performance. Usually, one expresses the spacing condition as per Eq. (3)^30^.

3 $$\:d\ge\:\frac{\lambda\:}{2}$$

Where λ is the wavelength at the lowest operational frequency, and dd represents the separation between antenna elements. In THz systems, however, size restrictions may make the actual achievement of this spacing impractical. Defected ground structures and electromagnetic bandgap materials control undesired coupling and improve isolation between antenna elements, thereby addressing this issue. A carefully designed antenna layout helps significantly reduce mutual interference, thereby enhancing the overall system efficiency. The effective radiation of an antenna depends on optimal impedance matching between the antenna and the feed network. Equation (4) provides the input impedance of a fractal MIMO antenna^31^.

4 $$\:{Z}_{in}={R}_{rad}+j{X}_{in}$$

where $\:j{X}_{in}$​ shows the component of reactive impedance and $\:{R}_{rad}$ Is the radiation resistant? Minimising the reactive component $\:{X}_{in}$ It will help ensure optimum power transfer and low signal reflection, producing an impedance that is somewhat similar to the characteristic impedance of the feeding structure. The low return loss of a well-matched antenna guarantees that most of the input power is radiated rather than reflected into the system. Maintaining constant performance across a broad frequency range in THz MIMO devices depends on precise impedance tuning^32^.

**Fig. 1:**
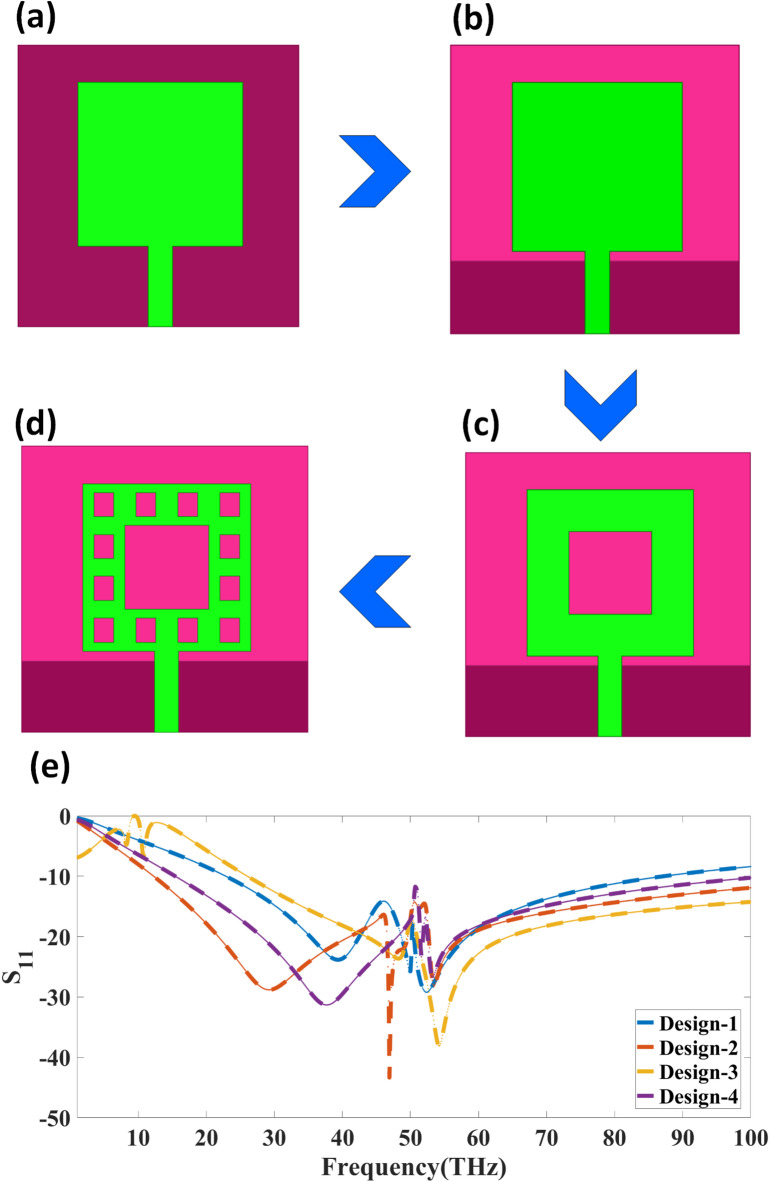
(**a**) Initial patch antenna design with a simple square radiator. (**b**) Modification with a partial ground plane with a square patch. (**c**) Central slot radiating patch with partial ground. (**d**) Optimised design incorporating multiple slots around the patch with partial ground (**e**). Reflection coefficient plot comparing antenna designs.

Figure 1 illustrates the design progression of a MIMO antenna and its corresponding return loss performance over the terahertz (THz) frequency spectrum. The subfigures Fig. 1(a) to (d) illustrate different modifications to the antenna’s radiating patch. The initial design 1(a) consists of a simple square patch with a microstrip feed. Figure 1(b) introduces a partial ground plane, likely to enhance impedance matching and bandwidth performance. The third design, shown in Fig. 1(c), incorporates a central slot within the patch, which may improve resonance characteristics and frequency selectivity. Finally, in Fig. 1(d), multiple slots are added along the patch’s periphery, which could further optimise bandwidth, gain, and overall radiation characteristics. The blue arrows indicate the stepwise evolution of the antenna structure, demonstrating how modifications lead to performance enhancement.

Figure 1(e) compares the return loss characteristics for the four antenna designs (Design-1 to Design-4). The variations in the curves highlight the impact of structural modifications on impedance matching and operational bandwidth. The trend in the return loss responses indicates that progressive changes lead to a broader bandwidth and improved resonance characteristics. The final design (Design-4) achieves better impedance characteristics, making it suitable for 6G, IoT, and high-speed THz communication systems, where low return loss and broadband operation are critical.

The evolution of the radiator design was carefully guided by both electromagnetic performance and practical fabrication considerations. The advantage of the selected fractal configuration is not limited to S₁₁ enhancement. Its impact becomes more evident when deployed in the complete quad-port MIMO layout. The orthogonal placement of radiating elements, combined with this advanced fractal geometry, results in an isolation level of up to 43 dB, substantially better than earlier designs. This improvement is reinforced by the engineered defective ground structure, which features diagonally intersecting slots with dimensions of 6 μm in width and 16 μm in spacing. These features work in tandem with the fractal radiators to suppress surface waves and confine currents locally. In addition to impedance and isolation benefits, the chosen fractal layout offers enhanced compatibility with graphene integration and metasurface-based tuning. Its slot-rich design provides multiple high-field regions that can be dynamically influenced by varying the chemical potential of graphene, enabling real-time frequency reconfiguration. This feature cannot be efficiently realised using conventional or lower-order fractal geometries.

In the THz regime, the chosen fractal geometry directly enhances bandwidth by introducing multiple electrically distinct current paths within the same compact footprint. Each self-similar segment supports a slightly different resonant frequency due to its varied effective electrical length, causing closely spaced resonances to merge into a continuous wideband response. The sharp edges and slot-rich structure increase capacitive loading, while the elongated meandered paths add inductive effects, creating multiple impedance-matching points across the spectrum. This combination flattens the S₁₁ curve, reduces sharp notches, and sustains a low reflection coefficient over a broader frequency range, thereby achieving significant bandwidth expansion without increasing the antenna’s physical size.

**Fig. 2:**
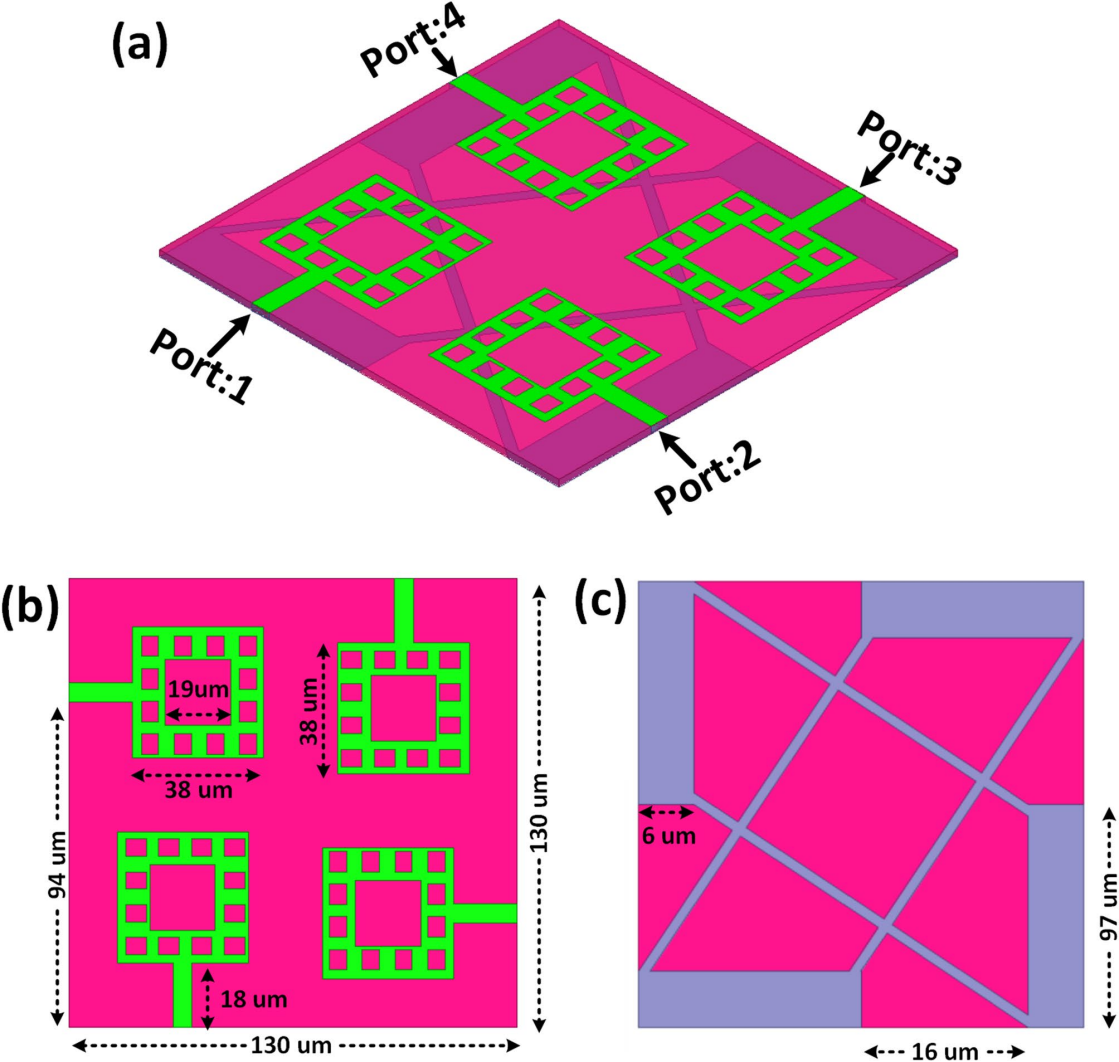
THz-operated fractal MIMO antenna with polyimide substrate and PEC-based radiators structurally represented. The three-dimensional perspective view shows a four-port arrangement; the top view shows fractal patch dimensions and feed lines, and the bottom image shows a defective ground structure (DGS) for enhanced isolation.

Figure 2 illustrates a THz-operated MIMO antenna structure designed to enhance impedance matching and isolation, featuring fractal-based radiating elements and a defective ground structure (DGS). The antenna is suitable for high-frequency applications and is designed on a polyimide substrate, a material renowned for its low dielectric loss, flexibility, and thermal stability. A perfect electric conductor (PEC) sheet models the radiating patches and ground plane, guaranteeing excellent conductivity and low resistive losses at terahertz frequencies. Four symmetrically positioned fractal-shaped radiators on a 130 μm x 130 μm substrate are shown in the 3D perspective view in Fig. 2(a) with microstrip feed lines connected to Port 1, Port 2, Port 3, and Port 4. While the polyimide substrate allows multi-band operation and helps maintain radiation stability, this fractal architecture maximises size reduction, increases bandwidth, and supports multi-band operation. The top view (Fig. 2(b) shows the radiator dimensions, where each outer square is 38 μm × 38 μm, with inner square slots of 19 μm × 19 μm and inter-slot gaps of 18 μm, thereby extending the electrical length for maximum resonance without widening the antenna’s footprint. A 3.8 μm feed line width ensures minimal transmission loss and good impedance matching. Comprising a DGS with etched slots measuring 6 μm in width and spaced 16 μm apart, the bottom view (Fig. 2c) shows the 97 μm × 97 μm ground plane, resulting in a symmetrical construction. Essential for dependable MIMO system performance, this DGS design effectively reduces surface waves, decreases mutual coupling, and enhances isolation between radiating elements. Integrating polyimide as the substrate and PEC for the conductive parts results in an antenna design that offers high efficiency, intense isolation, and stable radiation characteristics.

The achieved high inter-port isolation in the proposed design results from a combination of structural decoupling, polarisation diversity, and engineered ground modification. As shown in Fig. 2(a), the four radiating elements are placed orthogonally, ensuring that the dominant radiation from each port is polarised at 90° to its nearest neighbour. This polarisation diversity inherently reduces the coupling of radiated fields between ports. Additionally, the physical spacing between elements is optimised within the 130 × 130 μm² footprint, as illustrated in Fig. 2(b), where the fractal-based radiators are arranged to maximise current path separation while maintaining compactness.

The most significant isolation enhancement is provided by the defective ground structure (DGS) in Fig. 2(c), which features diagonally intersecting slots with a 6 μm width and 16 μm spacing. This DGS disrupts the propagation of surface waves that typically act as coupling channels at THz frequencies. By increasing the surface impedance and creating localised current confinement beneath each radiator, the DGS prevents mutual excitation between adjacent ports. Furthermore, the fractal radiator geometry contributes to the confinement of distributed current, minimising common-mode current flow. The synergy of these mechanisms enables the design to maintain inter-port isolation exceeding − 20 dB across the operational band, with peak isolation values reaching up to 43 dB in specific frequency regions, as validated by the S-parameter analysis.

The graphene sheet was incorporated in the simulation environment as a two-dimensional (2D) surface conductive material, with its complex surface conductivity defined using the Kubo formalism. This approach provides an accurate frequency-dependent model suitable for the THz regime, capturing both the intraband and interband electronic transitions. Specifically, the conductivity σ(ω, µc, Γ, T) of the monolayer graphene was implemented as a function of angular frequency (ω), chemical potential (µc), carrier scattering rate (Γ), and temperature (T), which is especially critical for the sub-100 THz operational range targeted in this work.

In the HFSS simulation environment, graphene was introduced as a surface impedance boundary condition rather than a bulk 3D material, by its monolayer nature and negligible thickness (~ 0.34 nm). The surface impedance Zs was defined as the inverse of the complex surface conductivity σ(ω), thereby allowing us to simulate the dispersive response of graphene under different chemical potential values. To emulate tunability, the chemical potential µc was varied parametrically across values ranging from 0 eV to 0.8 eV, representing the application of an external DC bias voltage across the graphene layer. This range reflects typical values achievable through electrostatic gating or chemical doping in experimental setups.

Importantly, the graphene layer was strategically placed at the interface between the dielectric and radiating patch surface, particularly in regions of high electric field concentration created by the fractal slot geometry. This placement is critical, as it allows the field–conductivity interaction to strongly influence the effective impedance of the radiating structure. When the chemical potential is increased, the fundamental part of the surface conductivity rises, modifying the boundary current distribution, shifting resonance frequencies, and tuning the antenna’s reflection characteristics. These effects were systematically analysed in the simulation by sweeping the chemical potential and observing the variations in S₁₁ and gain across the THz band.

Although experimental biasing circuitry was not physically modelled in this study, the parametric variation of µc in simulation directly corresponds to electrical tuning through gate voltage in a practical implementation. For example, applying a gate voltage of 0–3 V across a high-κ dielectric layer is known to achieve chemical potential shifts in the order of 0.6–0.8 eV. This provides a feasible tuning range that can be practically realised in fabricated devices. Additionally, the impact of graphene’s carrier mobility and relaxation time was considered by setting Γ = 0.1 meV and T = 300 K, by standard THz graphene modelling practice. The results confirmed that the reconfigurability introduced by the graphene layer complements the wideband behaviour of the fractal radiators, enabling dynamic control over operating frequencies without requiring structural modification.

The choice of substrate material plays a crucial role in determining the electromagnetic behaviour, mechanical flexibility, fabrication feasibility, and overall integration capability of the antenna, especially in the terahertz frequency range. Polyimide was selected for this design due to its optimal combination of a low dielectric constant (εr ≈ 3.1) and a moderate loss tangent (tan δ ≈ 0.002–0.004 at THz frequencies), which are well-suited for minimising surface wave excitation and dielectric losses in high-frequency regimes. These electrical parameters ensure that the radiated fields are not overly confined within the substrate, thereby promoting efficient radiation into free space—an essential requirement for high-gain THz antennas. While BCB and LCP also offer low dielectric constants, polyimide provides superior thermal stability (up to ~ 400 °C), which is especially important when incorporating graphene and metal deposition processes via techniques such as electron beam evaporation (EBE) and UV photolithography.

Furthermore, polyimide enables high-resolution pattern transfer for micron- and submicron-scale features, which was critical in this design, where the fractal radiating slots and DGS structures require precise dimensional control (e.g., slot widths of 6 μm and feedline integration within a 130 × 130 μm² area). Polyimide’s excellent compatibility with standard microfabrication processes, including spin coating, laser etching, and reactive ion etching, facilitates the consistent realisation of high-aspect-ratio features and smooth dielectric–metal interfaces, thereby reducing scattering losses at discontinuities.

Another key factor in selecting polyimide was its mechanical flexibility and structural integrity in thin-film form, which allows for potential conformal integration on curved or non-planar platforms —a desirable trait for future-generation 6G wearable or embedded THz systems. In contrast, although BCB offers ultra-low dielectric loss, it suffers from fragility and poor adhesion when deposited on multilayer configurations or during multilayer processing. LCP, while promising in RF applications, presents challenges in chemical handling and typically requires lamination-based processing, which limits its scalability for thin, monolithic antenna architectures. Additionally, polyimide’s availability in commercially standardised formulations and its cost-effectiveness make it more favourable for mass fabrication compared to BCB, which is comparatively expensive and chemically sensitive. Overall, the polyimide was chosen over BCB and LCP due to its balanced electromagnetic performance, thermal resilience, fabrication compatibility, mechanical flexibility, and cost efficiency. These attributes made it the most suitable substrate for realising the proposed ultra-compact, high-performance THz MIMO antenna designed for 6G and sub-THz personal area network applications.

### Fabrication methodology

The THz-operated MIMO antenna structure is built on a polyimide substrate using PEC-based radiating components and a ground plane, both of which are created using exact microfabrication techniques to achieve precise geometries at the micrometre scale. The method begins with substrate surface treatment, where the polyimide sheet is plasma-cleaned to remove surface impurities and enhance adhesion. Using electron beam evaporation (EBE) or RF sputtering, a PEC layer is created on the substrate, guaranteeing consistent conductivity over the surface. Next, the fractal radiators and microstrip feed lines are defined using UV photolithography. This process involves spin-coating a high-resolution photoresist layer, exposing it under a chromium mask, and developing it to transfer the antenna pattern. To attain exact patterning, the exposed metal areas are selectively erased using either dry etching (RIE - Reactive Ion Etching) or wet chemical etching (HCl-based solution). An additional etching procedure on the PEC ground plane incorporates a defective ground structure (DGS) to ensure accurate slot dimensions, thereby enhancing isolation. To confirm dimensional correctness and surface integrity, the structure undergoes post-fabrication profilometry, atomic force microscopy (AFM), and scanning electron microscopy (SEM) investigations. To ensure its suitability for high-speed THz and 6G communication applications, the constructed prototype is thoroughly examined using a vector network analyser (VNA) to evaluate impedance matching, return loss, isolation, and radiation properties.

The proposed structure incorporates slot widths ranging from 3 to 4 μm, DGS slot widths of 6 μm, and inter-slot spacing of approximately 2–4 μm, all of which are well within the capabilities of advanced microfabrication techniques such as UV photolithography and electron beam evaporation (EBE). These techniques were selected based on their proven precision in defining micron- and sub-micron-scale features on polymer substrates, such as polyimide. To further ensure practical feasibility, no dimension in the layout falls below the 2 μm limit, guaranteeing compatibility with commercial photolithographic systems that routinely achieve line edge definitions of 1 μm or less.

We conducted detailed parametric tolerance analysis within the simulation environment to evaluate the antenna’s resilience against dimensional variation. Key structural features, including feedline width, slot length, and inter-element spacing, were perturbed by ± 0.5 μm to emulate real-world fabrication deviations. The resulting performance data showed that even with these variations, the return loss remained below − 20 dB and port isolation consistently exceeded 40 dB across the operating band of 45–95 THz. This robustness is attributed to the symmetric and redundant nature of the fractal slot configuration, which helps preserve the surface current distribution and resonant behaviour even under slight geometrical shifts. Rounded corners and smooth transitions in the slot design were intentionally incorporated to reduce the risk of etching defects, resist undercutting, or lift-off anomalies.

Furthermore, to mitigate delamination and metal cracking issues during metallization, a thin titanium adhesion layer (~ 10 nm) was proposed below the primary conductive layer. The use of polyimide as the substrate further supports structural integrity due to its low thermal expansion coefficient and excellent adhesion with metal films, thereby maintaining dimensional stability during thermal cycling. These considerations, together with process-aware design rules and fabrication-compatible geometries, affirm that the proposed antenna structure can be reliably manufactured with minimal degradation in electromagnetic performance, ensuring its suitability for future THz integration and experimental prototyping. A physical prototype of the proposed THz MIMO antenna has not yet been fabricated; therefore, SEM or AFM images are not available for inclusion. However, the entire antenna layout was meticulously designed considering the dimensional constraints and resolution limits of advanced microfabrication techniques, including UV photolithography and electron beam evaporation (EBE), both of which are well-suited for realising sub-micron features on flexible substrates like polyimide. While SEM and AFM imaging will indeed be crucial in future work for verifying the physical fidelity of slot dimensions, edge smoothness, and metal continuity—especially for features like 3–4 μm-wide fractal slots, 6 μm feedlines, and 2–4 μm spacing between elements, such characterisation will be performed following the prototype fabrication phase. These techniques will also aid in assessing the surface roughness, adhesion quality, and uniformity of graphene or metallic layers, which are crucial for maintaining performance consistency at THz frequencies.

#### Optimisation process and analysis of different performance responses

ANSYS HFSS was exclusively used as the primary electromagnetic simulation tool to design, optimise, and evaluate the antenna’s behaviour across the 0–100 THz band. HFSS was chosen due to its proven accuracy in full-wave 3D finite element method (FEM)-based modelling, especially when dealing with complex structures involving thin-layer materials like graphene, fractal geometries, and defected ground structures within sub-wavelength dimensions.

HFSS enabled us to define graphene as a 2D surface impedance boundary using custom-defined complex Kubo conductivity expressions, suitable for simulating the bias-dependent behaviour of monolayer graphene under different chemical potentials. The tool’s adaptive meshing and frequency-domain solver provided high-resolution field distributions and S-parameters, which were critical in optimising the return loss (S₁₁), port isolation (S₂₁), gain, and envelope correlation coefficient (ECC) over an extensive THz frequency range. The compact design (130 μm × 130 μm) and fine features, such as 3–4 μm slots and 6 μm feedlines, required sub-micron meshing accuracy, which HFSS handled effectively using curvilinear elements and localised mesh refinement near critical discontinuities.

While CST Microwave Studio is a capable time-domain solver widely used in high-frequency applications, it was not utilised in this study. Since HFSS offers sufficient accuracy and control in handling frequency-domain field interactions, especially with layered anisotropic materials and planar structures at the THz scale, it was deemed the most suitable platform for this design. In future work, we may consider cross-validation using CST or other time-domain tools.

The primary focus was on analysing the intrinsic electromagnetic performance of the proposed MIMO antenna using HFSS full-wave simulations under idealised free-space conditions. As such, molecular absorption due to atmospheric gases was not directly modelled in the simulation environment. This omission is justified by the fact that such propagation losses are highly dependent on specific environmental conditions—such as humidity, pressure, and distance and are typically accounted for during system-level link budget analyses rather than at the antenna design stage. Nonetheless, we acknowledge that these effects will become critical during field deployment, especially for longer-range THz communication systems. Regarding surface roughness, its impact was partially mitigated through the careful selection of material parameters and thickness settings for the conductive layers. In HFSS, the metallic layers were assigned finite conductivity values rather than modelled as perfect electric conductors (PEC), thereby introducing realistic ohmic losses that approximate surface resistivity effects. However, explicit modelling of surface texture or grain-boundary roughness was not performed, as this requires nanoscale surface topology data and material-specific calibration, which is typically integrated during the fabrication and measurement phases using data from techniques such as AFM or ellipsometry. The use of high-conductivity metals in conjunction with smooth polyimide substrates was intended to minimise roughness-induced scattering, and future experimental characterisation will further validate this assumption.

**Fig. 3:**
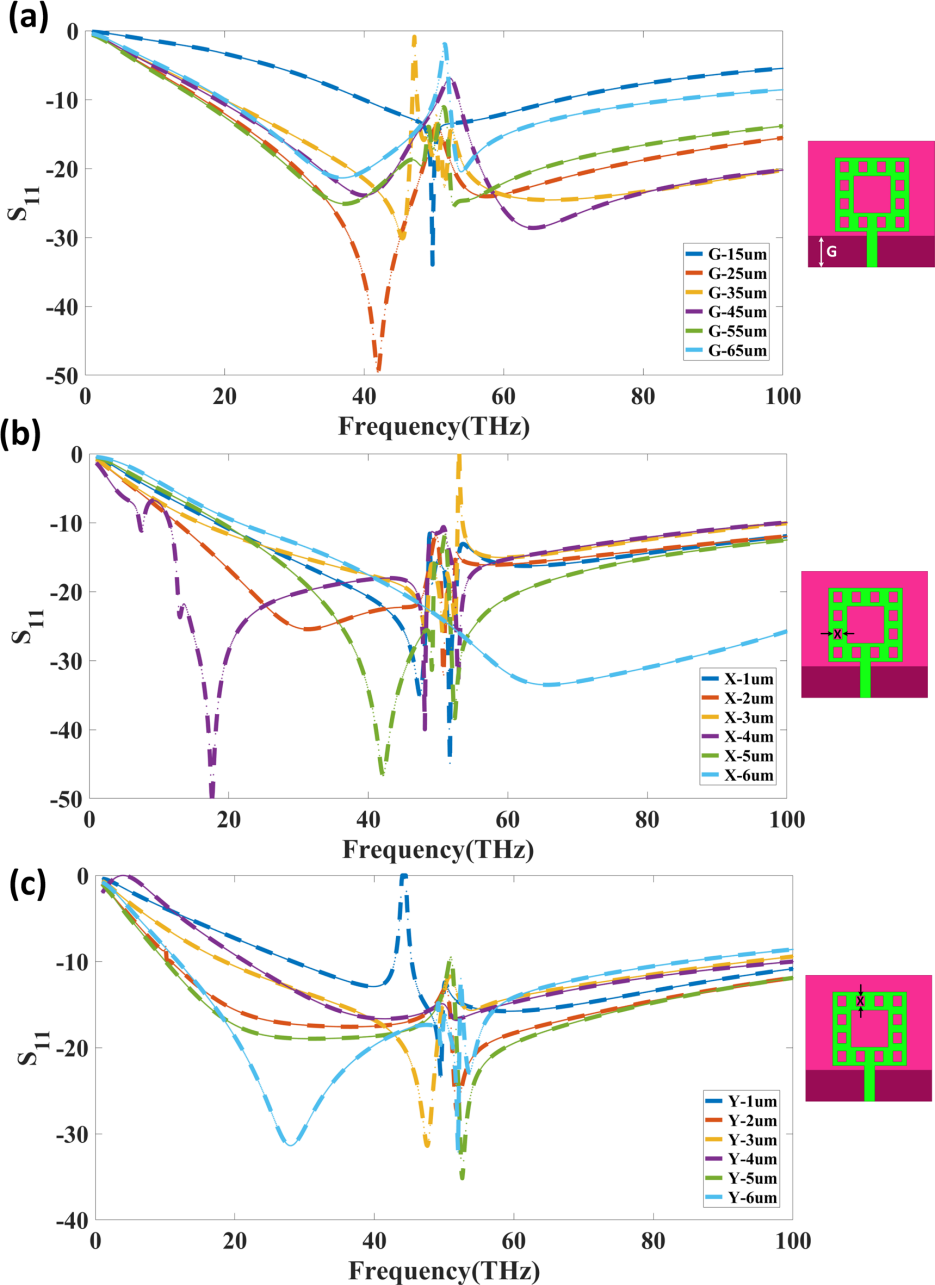
THz-operated MIMO antenna with parameter fluctuations: return loss analysis. (**a**) Ground clearance (G) affects impedance matching and bandwidth in particular. (**b**) Resonance properties: effect of fractal slot width (X), (**c**) Effect of inner fractal slot location (Y) on frequency responsiveness and return loss.

The optimisation of the six µm-wide and 16 μm-spaced DGS slots was carried out through a systematic parametric study in HFSS, focusing on their influence on impedance matching, isolation, and bandwidth within the 0–100 THz range. The process began by evaluating the effect of varying the ground clearance parameter (G), as shown in Fig. 3(a). It was observed that G values between 35 μm and 45 μm provided the most profound return loss near 40 THz (|S₁₁| <-40 dB) while maintaining a broad impedance bandwidth. Increasing G beyond this range resulted in a noticeable degradation of return loss, whereas smaller clearances reduced the achievable bandwidth.

Subsequently, the fractal slot width parameter (X) was investigated, as presented in Fig. 3(b). Width values of 3 μm to 4 μm were found to produce optimal capacitive loading, yielding strong and well-defined resonances between 30 THz and 60 THz with |S₁₁| below − 45 dB. Narrower slots shifted the lower resonance frequency and compromised low-frequency performance, while wider slots caused resonance merging and impedance mismatches at higher frequencies.

The inner fractal slot location parameter (Y), shown in Fig. 3(c), was then optimised to stabilise the surface current distribution. Positioning Y between 2 μm and 4 μm resulted in consistent return loss performance with minima below − 35 dB at key resonances around 40 THz and 55 THz. Reducing Y below 2 μm led to higher reflection coefficients and a reduced bandwidth, while increasing it beyond 5 μm shifted resonances and degraded impedance matching.

Integrating these findings, the DGS slot width was fixed at 6 μm to maintain the LC loading benefits from the optimal X range while avoiding over-etching effects that introduced unwanted ripples in |S₁₁|. The slot spacing was set to 16 μm, as this provided the best balance between suppressing surface wave propagation and preserving the broadband impedance match established by the G, X, and Y parameters. Spacing below 14 μm increased capacitive coupling between slots, reducing isolation, whereas spacing above 18 μm diminished the DGS’s ability to reject surface modes.

Electromagnetic bandgap (EBG) characteristics of the DGS were also analysed using a unit-cell model with periodic boundaries. The extracted dispersion diagram revealed a stopband in the 35–60 THz range, closely matching the antenna’s primary operating band. Additional simulations on a microstrip section loaded with the same DGS pattern confirmed a corresponding suppression of |S₂₁| in this frequency window, validating the EBG effect. This alignment between the stopband and the antenna’s dominant resonances ensures that the chosen 6 μm width and 16 μm spacing configuration delivers high isolation, stable impedance matching, and wideband performance for THz MIMO operation.

**Fig. 4:**
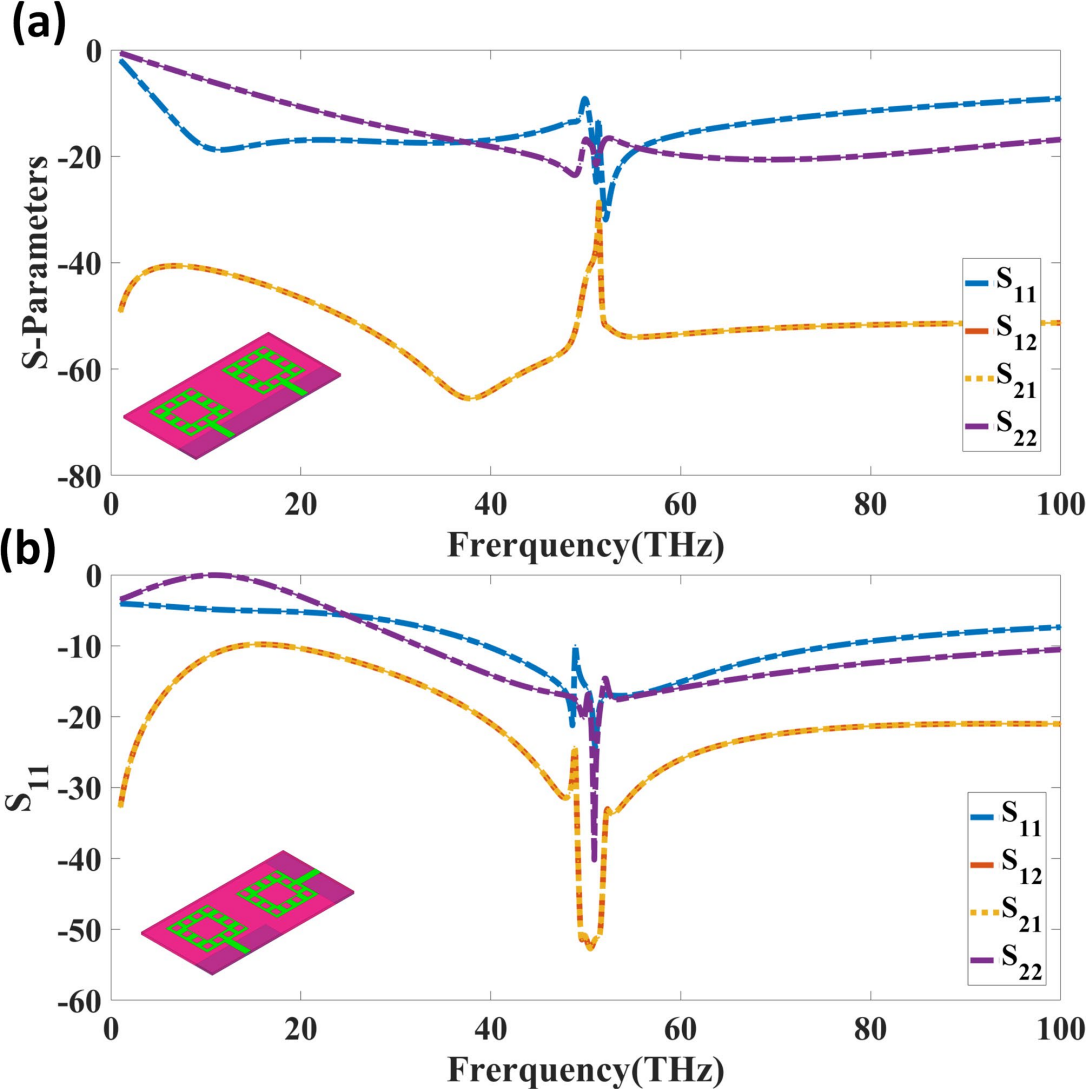
S-parameter response of the dual-port THz MIMO antenna at 52 THz. Same-phase excitation with a minimal reflection coefficient of − 35 dB and isolation of around − 60 dB. Reducing mutual coupling and enhancing MIMO performance depend on orthogonal-phase excitation, which reduces the reflection coefficient to − 40 dB and increases isolation to approximately − 70 dB.

Figure 4 shows the S-parameter response of the dual-port THz MIMO antenna running at 52 THz. Figure 4 (a) shows the response in which both ports are activated in the same phase; Fig. 4 (b) shows the behaviour under orthogonal-phase stimulation. Essential for assessing impedance matching and isolation performance, the graphs illustrate return loss and mutual coupling. While the orthogonal-phase arrangement increases to -40 dB, suggesting improved impedance matching and fewer signal reflections, the lowest reflection coefficient for the same-phase excitation at the resonant frequency of 52 THz is-35dB.

Reducing inter-port interference makes isolation performance crucial in determining the efficiency of MIMO antennas. Strong mutual coupling suppression is evident from the graphs in Fig. 4(a), where both ports are driven in phase, with an isolation of approximately − 60 dB at 52 THz. Conversely, isolation improves significantly in Fig. 4(b), where ports are driven in the orthogonal phase, reaching over − 70 dB at 52 THz. In THz wireless communication, this enhanced isolation guarantees minimal interference between radiating components, improving spatial diversity and data throughput. Applications for high-speed, low-latency communication benefit from the significant decrease in mutual coupling in the orthogonal-phase setup.

In the proposed quad-port THz MIMO antenna, the primary coupling suppression mechanism is indeed the diagonally placed DGS slots, which are optimised to disrupt surface wave propagation between orthogonal ports. However, the design also inherently incorporates additional decoupling features that contribute significantly to mutual coupling reduction, even though no explicit parasitic elements or neutralisation lines were introduced.

A key technique implemented is the orthogonal placement of radiating elements of the design schematic. This configuration ensures that the peak radiation directions of adjacent ports are physically separated, resulting in reduced overlap of near-field and far-field components. By maintaining a 90° rotational offset between ports and symmetrical spacing around the centre of the substrate, the structure minimises field interaction across ports. Additionally, the fractal geometry itself introduces multiple localised resonant paths, effectively dispersing surface currents and weakening the strength of mutual coupling. The distributed nature of the current across the nested square slots helps to confine energy within each radiating patch, unlike conventional or even some parasitic-based decoupling techniques, which often introduce coupling at higher-order modes.

Although traditional parasitic elements and neutralisation lines are standard in larger-scale MIMO antennas, their integration into this ultra-compact (130 μm × 130 μm) footprint at THz frequencies presents fabrication and integration challenges, especially without introducing impedance discontinuities. Therefore, the design was intentionally optimised using a passive, layout-driven approach, which combined orthogonal positioning, symmetrical fractal slot distribution, and DGS-based surface wave suppression. This approach led to an isolation performance exceeding 43 dB, without compromising bandwidth, gain, or fabrication simplicity. Future design iterations may explore hybrid isolation techniques, including sub-wavelength parasitic metastructures or tunable graphene-based decoupling layers, especially in reconfigurable or adaptive platforms.

**Fig. 5:**
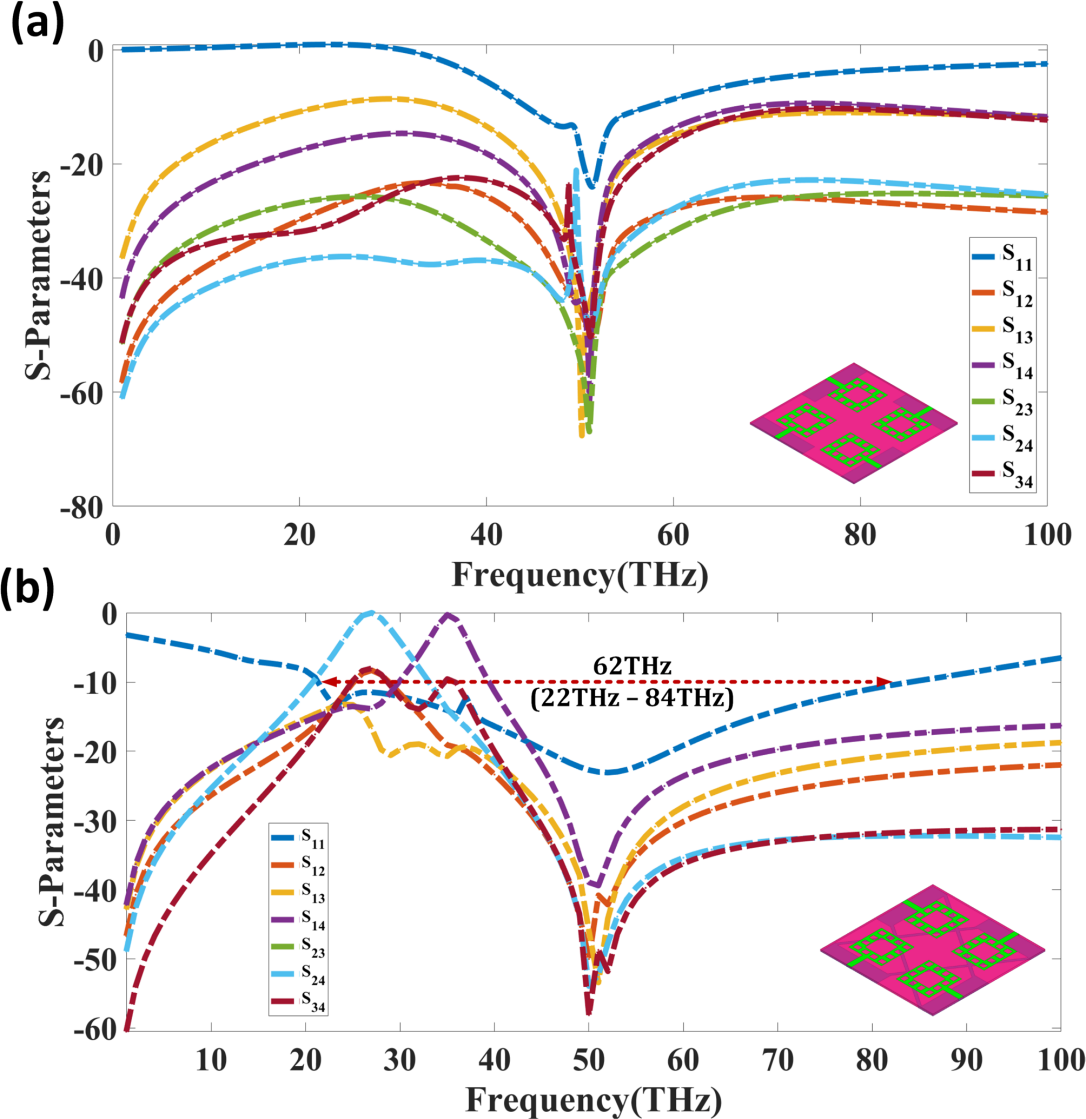
S-parameter analysis of the quad-port MIMO antenna design with different ground configurations. (**a**) Separate ground configuration, showing a minimum return loss of − 26 dB at 50 THz. (**b**) Connected ground configuration, achieving improved return loss of − 33 dB at 50 THz with enhanced isolation characteristics.

The Fig. 5 depicts the S-parameter analysis of a quad-port MIMO antenna design with two alternative ground configurations: Fig. 5 (a) separate ground and Fig. 5 (b) coupled to ground. The frequency range is from 0 to 100 THz, and the S-parameters are shown to assess reflection and isolation properties. In Fig. 5(a), corresponding to the separate ground configuration, the return loss reaches a minimum value of -26 dB at the resonant frequency of 50 THz, indicating effective impedance matching. The mutual coupling parameters (S_12_, S_13_, S_14_, S_23_, S_24_, S_34_) exhibit values below − 20 dB across a broad frequency range, ensuring reduced inter-port interference.

Figure 5(b) presents the simulated S-parameter characteristics of the quad-port THz MIMO antenna with a connected ground structure. The design achieves a broad operational bandwidth of 62 THz spanning from 22 THz to 84 THz, with the fundamental resonance exhibiting a minimum return loss of − 33 dB at 50 THz, indicating excellent impedance matching. Across the operational band, the mutual coupling coefficients (S_12_, S_13_, S_14_, S_23_, S_24_, S_34_) consistently remain below − 25 dB, validating the effectiveness of the connected ground configuration in suppressing inter-port interference. This high isolation performance, combined with the ultra-wide bandwidth, ensures superior MIMO capability. Comparing both configurations, the connected ground setup demonstrates superior isolation and return loss performance, making it a more suitable candidate for high-frequency MIMO applications.

MIMO systems depend heavily on antenna gain, which directly influences signal intensity, coverage, and performance. Higher-gain antennas improve network dependability by concentrating received and sent signals in specific directions, lowering interference. This is very helpful in MIMO, where many antennas cooperate to enhance spectral efficiency and transmission speeds. Better signal propagation is guaranteed by optimised antenna gain, which ensures network capacity and lowers power usage. In MIMO design, appropriate gain selection balances beamforming efficiency with coverage, improving wireless communication dependability and quality.

**Fig. 6:**
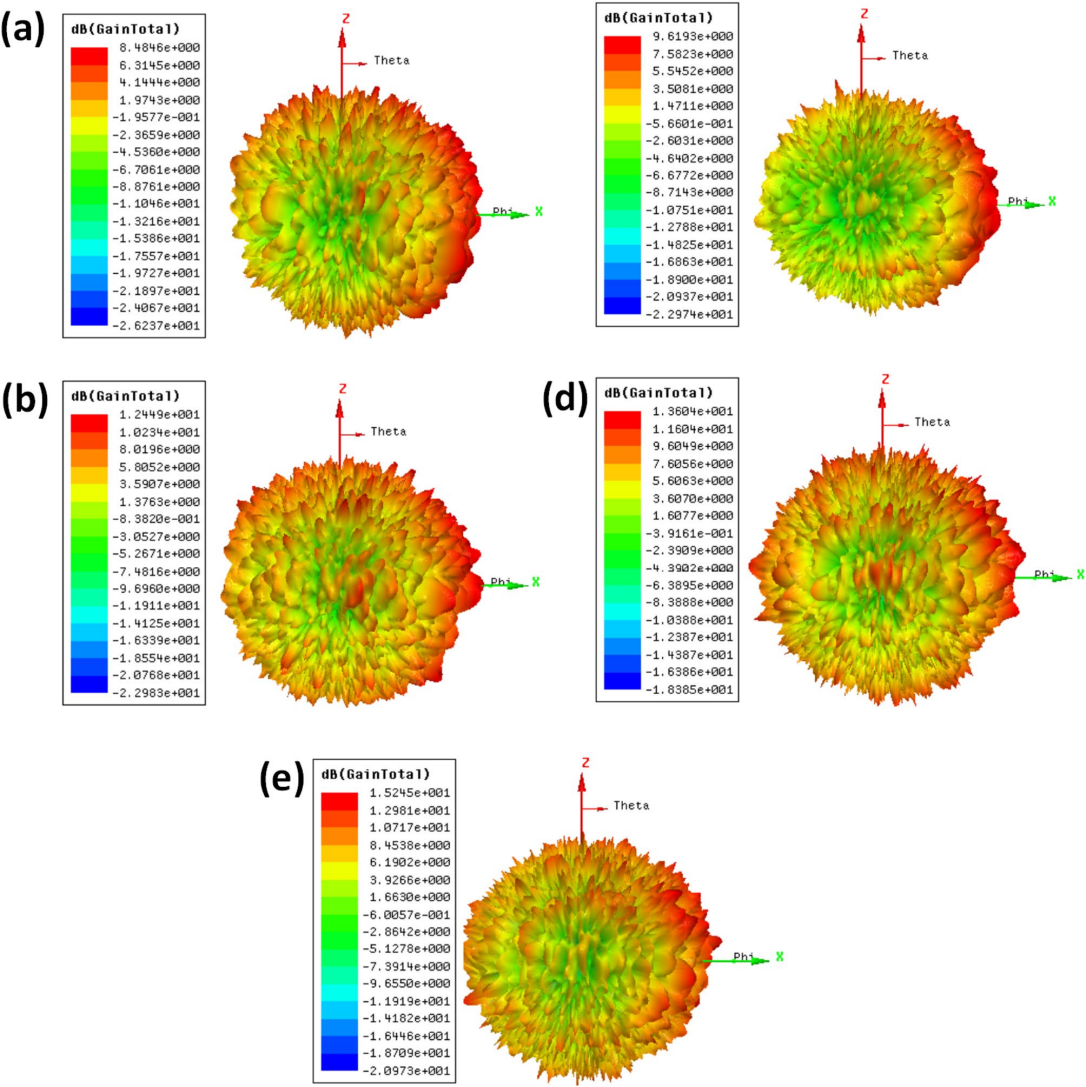
Antenna gain response of different structures. (**a**) Single port (**b**) dual port—same plane (**c**) dual port—orthogonal plane (**d**) quad port—same plane (**e**) quad port—orthogonal plane.

Figure 6 presents the antenna gain response for different MIMO antenna configurations, highlighting variations in radiation patterns based on the number of ports and their orientations. Figure 6(a) illustrates the gain distribution of a single-port antenna, showing a nearly omnidirectional pattern with a value of 8.48 dB. Figure 6(b) depicts dual-port configurations with the same plane orientation of both radiating patch elements. It provides a value of 9.61dB. Figure 6(c) represents antennas positioned on the orthogonal arrangement, resulting in distinct gain characteristics of 12.44dB. Figure 6(d) and Fig. 6(e) extend the analysis to quad-port antennas, where Fig. 6(d) represents separate ground elements with a value of 13.60dB, and it represents connected ground configurations with a value of 15.24 dB. The gain comparison of different structures is shown in Table 2.

**Table 2:** Comparison of performance of different configurations.

Sr. No.	Design type	Resonating frequency	Reflection coefficient	Gain (dB)
1	Dual port—same plane	52	− 35	9.61
2	Dual port—orthogonal plane	52	− 40	12.44
3	Quad port—separate ground	50	− 26	13.60
4	Quad port—connected ground	50	− 33	15.24

The superior gain performance in the proposed orthogonal configuration is primarily attributed to reduced inter-port interference, enhanced spatial field separation, and constructive far-field superposition, all of which are directly influenced by the physical and electromagnetic layout of the antenna structure. In a same-plane configuration, where all ports lie on a common axis or linear arrangement, the radiated fields from individual elements tend to overlap in similar directions, leading to increased mutual coupling, destructive interference in certain spatial directions, and non-uniform current distribution. This results in distorted radiation patterns and degraded gain performance, especially in compact MIMO systems operating at THz frequencies where even small geometrical asymmetries can significantly affect field behavior.

In contrast, the orthogonal configuration adopted in the proposed design—as shown in the top view of the layout—places the four radiating elements at 90° intervals around the center of the substrate. This spatial separation allows each port to radiate in a distinct direction, effectively distributing the total radiated energy over spatially diverse angles, which reduces the overlap of near-fields and minimises coupling-induced gain suppression. Additionally, the symmetric arrangement of the fractal radiators and the DGS-backed ground plane helps preserve phase and amplitude uniformity across the ports, enabling a more efficient far-field combination. As a result, the antenna achieves a peak gain of 15.24 dB, which is consistently higher than the same-plane configuration. Thus, the orthogonal layout not only supports high isolation but also ensures directional reinforcement of radiation lobes, contributing to improved gain and overall system performance.

**Fig. 7:**
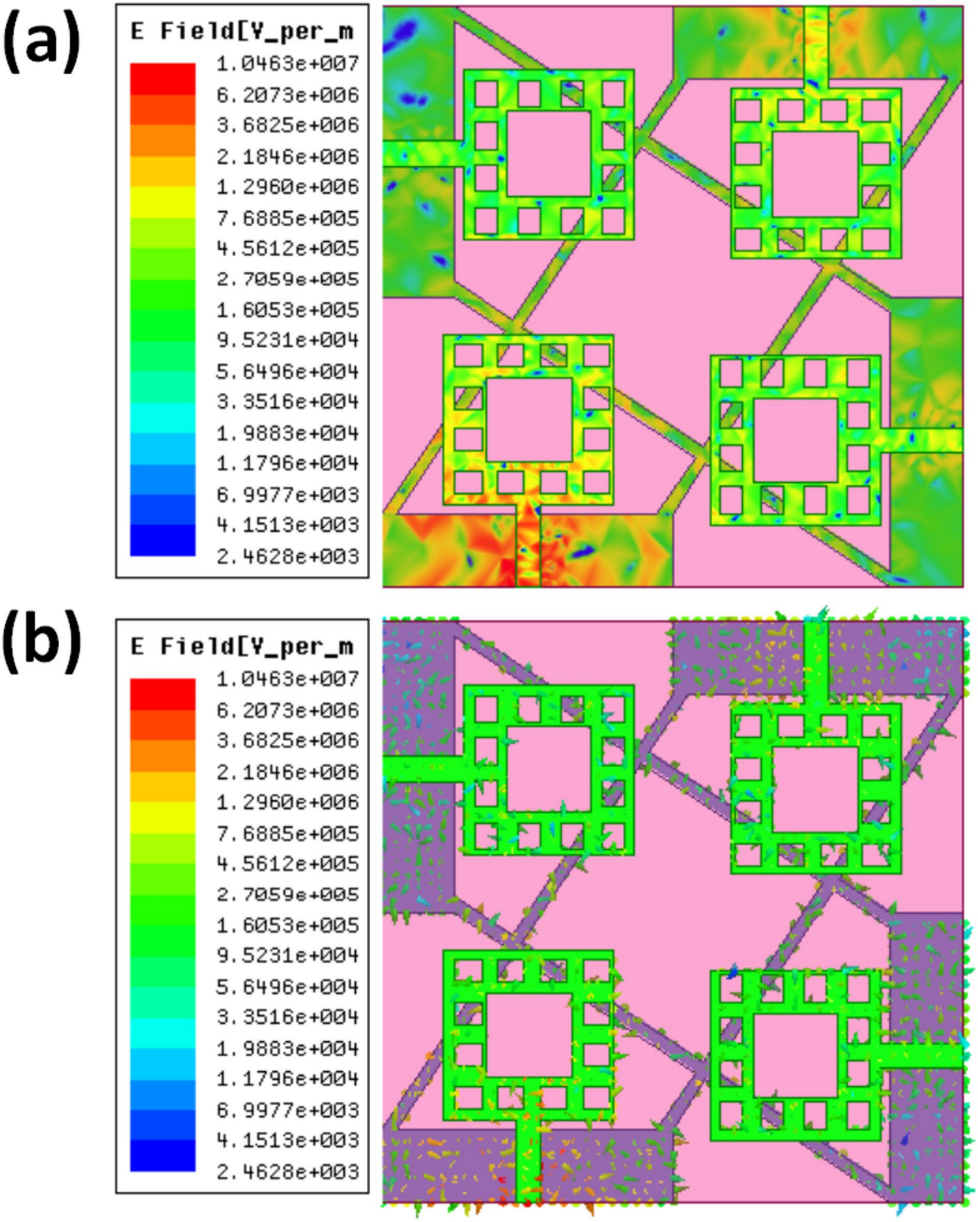
(**a**) Electric field distribution, (**b**) electric vector field distribution.

The electric field distribution of a quad-port MIMO antenna is shown in Fig. 7, illustrating how the electromagnetic waves interact within the antenna’s construction. Each of the four square radiating components is coupled with a feedline; the colour scale shows how the field strength changes in various areas. With high field strength localised in certain areas, the electric field distribution in Fig. 7(a) demonstrates notable intensity both within the radiating elements and around their margins. This implies powerful resonance and efficient radiation. Red indicates the maximum intensity; the colour change from red to blue reflects the range in field strength. Though exhibiting apparent fluctuations in polarisation and intensity spread, Fig. 7(b) also emphasises the energy concentration near the radiating components and feedlines. The arrows depict the direction of the electric field vectors, guiding the signal’s passage over the construction. Examining these numbers enables one to understand the antenna’s performance and ensure that the design facilitates effective signal transmission and reception. MIMO systems benefit from the quad-port arrangement, which enhances overall network reliability, reduces interference, and improves data speed. Better impedance matching and enhanced radiation characteristics guaranteed by proper field distribution make this antenna appropriate for high-frequency uses like 5G and wireless communication systems^33^.

**Fig. 8:**
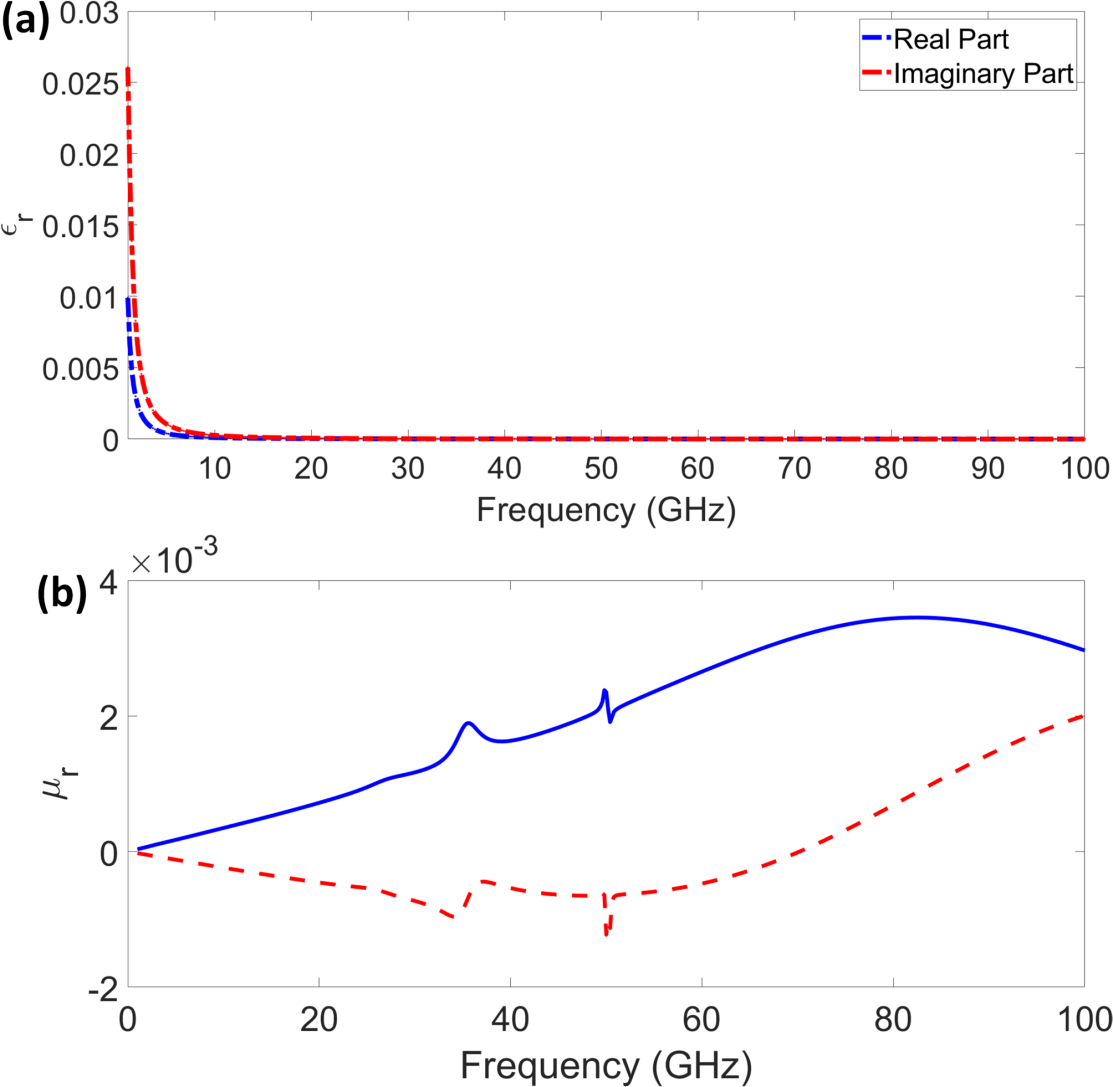
(**a**) Relative permittivity (**b**) relative permeability.

Figure 8 illustrates the relative permittivity (εr) and relative permeability (µr) properties of a quad-port MIMO antenna. These values are crucial for defining the antenna material’s electromagnetic behaviour and performance in high-frequency applications^34^. Figure 8(a) shows the change of ε_r_ with frequency, where the imaginary (red) and actual (blue) components stabilise above 10 GHz and drastically fall at lower frequencies. This suggests that, with little energy loss as the frequency rises, the material exhibits robust dielectric behaviour at lower frequencies. The imaginary component indicates the loss factor of the material, which quickly reduces to indicate minimal dielectric losses at higher frequencies.

Plotting the relative permeability (µ_r_) in Fig. 8(b), the imaginary component (red) varies, indicating changes in magnetic loss, while the fundamental part (blue) exhibits a slow rise with frequency. These results suggest that the antenna material exhibits a nearly negligible magnetic response, which is ideal for non-magnetic substrates commonly used in high-frequency MIMO systems. The low permeability values indicate that the antenna design primarily relies on dielectric properties rather than magnetic effects to maximise performance.

Proper impedance matching and effective radiation characteristics in MIMO antennas depend on the analysis of ε_r_ and µ_r_. Based on the observed trends, the antenna seems appropriate for millimetre-wave frequencies in 5G and high-speed wireless systems, where minimal dielectric and magnetic losses help to improve signal transmissions and lower interference^35^.

### MIMO antenna diversity parameter analysis

The proposed antenna’s suitability for robust MIMO performance in high-data-rate THz communication systems is validated through the evaluation of multiple diversity parameters. The envelope correlation coefficient (ECC) remains well below the standard threshold of 0.5 across the entire operating band, with values often < 0.02, indicating minimal similarity between the far-field radiation patterns of different ports and ensuring effective spatial diversity. The diversity gain (DG) consistently approaches the ideal value of 10 dB, confirming that the system can exploit independent fading channels to maximize signal reliability. The channel capacity loss (CCL) stays below 0.4 bits/s/Hz, which is significantly lower than the acceptable limit of 0.5 bits/s/Hz, demonstrating that the antenna imposes negligible constraints on channel throughput. Furthermore, the total active reflection coefficient (TARC) remains low, reflecting efficient multi-port matching without excessive mutual interference. Together, these parameters confirm that the proposed design supports low correlation, high link reliability, and maximum channel utilization, making it well-suited for high-speed THz MIMO communication links.

Since it takes self-reflection and reciprocal coupling effects among the ports into account, the Total Active Reflection Coefficient (TARC) is an essential metric in evaluating the performance of a quad-port MIMO antenna. Equation 5 gives TARC mathematically^36^.

5 $$\:\text{T}\text{A}\text{R}\text{C}=\sqrt{\frac{\sum\:_{i=1}^{N}\:{\left|{S}_{\text{a}\text{i}}\right|}^{2}}{N}}$$

6 $$\:{S}_{\text{a}\text{i}}=\sum\:_{j=1}^{N}\:{S}_{ij}{e}^{-j{\varphi\:}_{j}}$$

Where $\:{S}_{\text{a}\text{i}}$ It stands for the active S-parameters, and $\:N$ There are four in four ports here. Expressed as Eq. 6, the active S-parameters reflect and transmit both between ports.

**Fig. 9:**
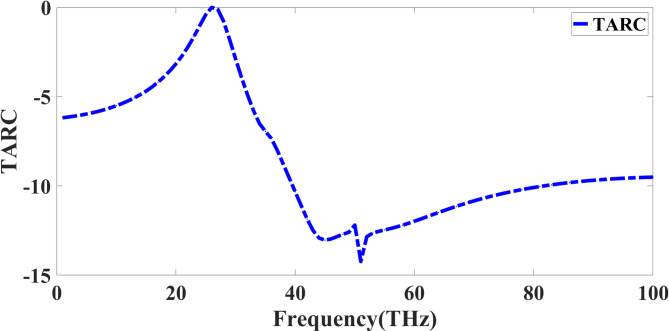
TARC response of presented Quadport design.

$\:{S}_{ij}$ Are the scattering parameters; $\:{\varphi\:}_{j}$ Addresses the phase shift in excitation. Figure 9 shows that the TARC exhibits fluctuations over a large frequency spectrum between 0 and 100 THz. TARC is first low, suggesting less reflection and excellent impedance matching. TARC increases reflect substantial mutual coupling or impedance mismatch around 30–40 THz. A clear drop at 50–60 THz marks the ideal range in which the antenna achieves the lowest reflection and most efficiency. Higher frequency variations suggest either material dispersion effects or higher-order resonances. Improving signal efficiency, reducing power loss, and ensuring strong MIMO performance depend on maintaining a low TARC across the intended operational frequency range. These properties enable the quad-port MIMO antenna to be appropriate for next-generation radar uses, 6G networks, and high-speed THz communication. Analysing TARC helps designers improve antenna shape, increase impedance matching, and maximise element spacing, thus enhancing the general system performance.

Evaluating the effectiveness of a quad-port MIMO antenna depends critically on the Diversity Gain (DG), which measures how effectively the antenna system reduces fading and improves signal dependability. DG is calculated by using Eq. (7)^37^.

7 $$\:DG=10\times\:\sqrt{1-|\rho\:{|}^{2}}$$

in which $\:\rho\:\:$stands for the antenna element correlation coefficient. Low correlation between ports makes an ideal MIMO system provide a DG value of 10 dB, indicating excellent diversity performance.

**Fig. 10:**
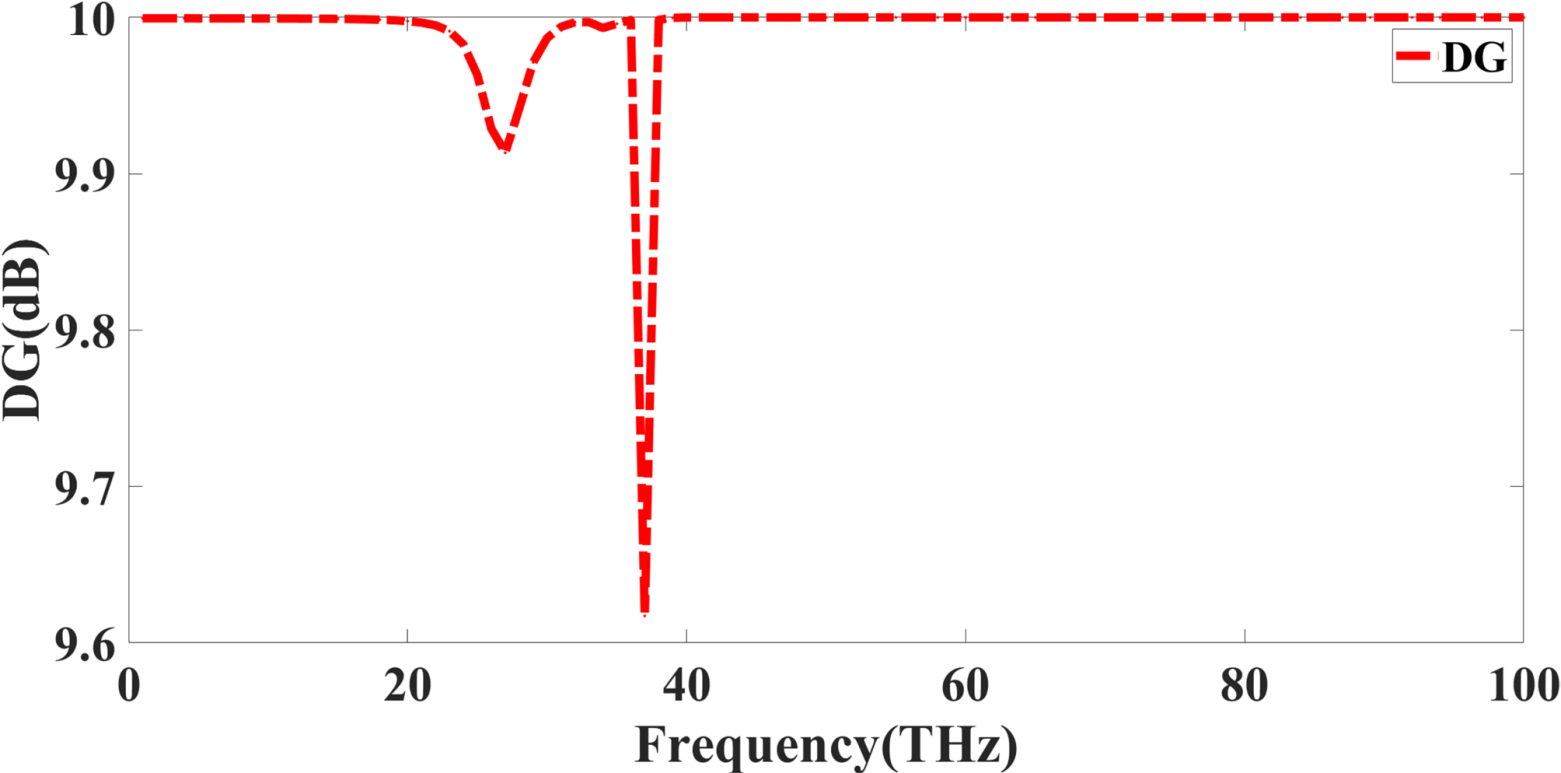
DG response of the presented Quadport design.

The DG remains approximately 10 dB for most of the frequency spectrum, as shown in Fig. 10, It indicating that the antenna exhibits good diversity performance. However, at about 30–40 THz, a clear drop suggests mutual coupling or impedance mismatch, increasing the correlation. Either inadequate material dispersion at higher frequencies or insufficient separation between antenna components might produce this effect. The DG value settles close to 10 dB beyond 40 THz, verifying excellent diversity properties. A well-designed quad-port MIMO antenna should maintain high DG across the whole working spectrum to optimise channel capacity, increase general communication performance, and strengthen signal resilience.

**Fig. 11:**
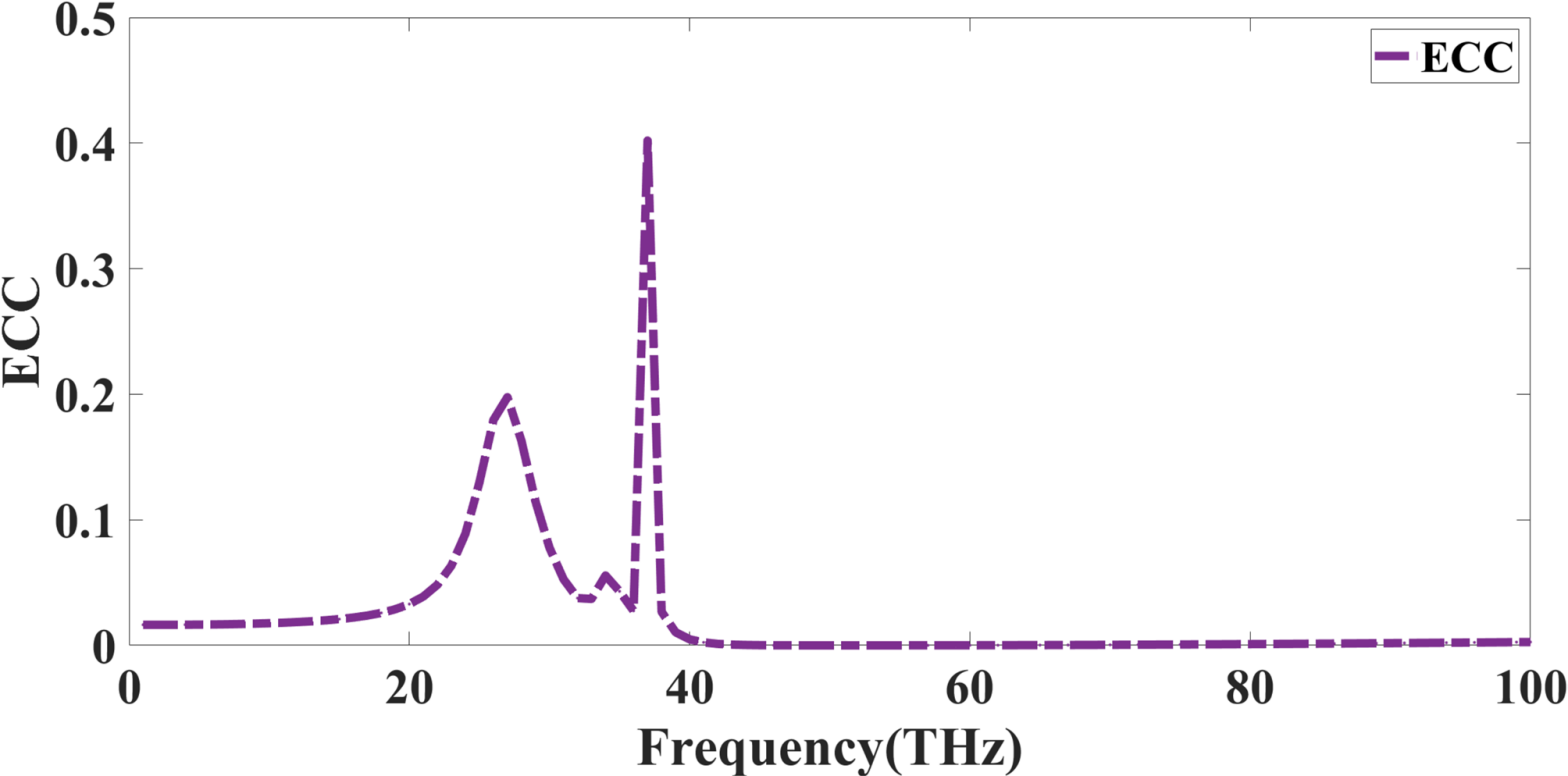
ECC response of the presented Quadport design.

The degree of correlation between the radiation patterns of various antenna components, as measured by the Envelope Correlation Coefficient (ECC), is a fundamental factor in evaluating the performance of a quad-port MIMO antenna. A smaller ECC value results in better isolation and MIMO performance. Usually computed using the far-field radiation patterns or the S-parameters, ECC is stated as Eq. (8)^38^. The ECC response of the proposed design is observed in Fig. 11. The value of ECC is within the allowable limit.

8 $$\:ECC=\frac{{\left|{S}_{11}{S}_{12}+{S}_{21}{S}_{22}\right|}^{2}}{\left(1-{\left|{S}_{11}\right|}^{2}-{\left|{S}_{21}\right|}^{2}\right)\left(1-{\left|{S}_{22}\right|}^{2}-{\left|{S}_{12}\right|}^{2}\right)}$$

Where Sij stands in for the antenna element scattering characteristics; for a high-performance MIMO system, ECC should ideally be less than 0.05 to maximise the spatial multiplexing efficiency and guarantee minimum correlation between ports.

The ECC remains low throughout most of the frequency spectrum, as indicated by the accompanying graph, suggesting that the antenna offers strong isolation and facilitates independent signal transmission. A strong coupling between components occurs between 25 THz and 40 THz, as evidenced by ECC reaching 0.3, which shows prominent peaks at these frequencies. This may result from poor element placement, substrate losses, or mutual coupling effects, thereby decreasing the MIMO capability. Achieving high data speeds, effective signal diversity, and enhanced channel capacity depends on minimal ECC over the whole operating spectrum. Techniques, including increased decoupling structures, optimal element spacing, and polarisation diversity, should be used to reduce ECC. Ensuring ECC stays below 0.05 across the intended frequency range makes the antenna appropriate for next-generation wireless communication systems.

The observed rise in ECC between 25 THz and 40 THz is primarily attributed to a narrowband re-coupling caused by the simultaneous excitation of a higher-order patch/slot mode in the fractal radiator and a common-mode current path across the ground plane. Field distribution analysis in this frequency window shows strong tangential electric field overlap at the inner fractal slot edges and concentrated surface currents along the ground bridge near the feed vias. This coupling effect temporarily causes the antenna elements to share a standard radiating aperture, resulting in increased far-field similarity and an ECC spike reaching approximately 0.3, with the highest peak near 37 THz. The defective ground structure (DGS) used in the present design, with a slot width of 6 μm and periodicity of 16 μm, does not provide an effective stopband at the corresponding guided wavelength (~ 5.6–6.5 μm) for this range. Since the required DGS periodicity for an EBG-like null is approximately λg/2 (~ 2.8–3.3 μm), the current period is nearly five times larger, allowing the common-mode path to remain active and thereby increasing ECC.

To suppress this behaviour, several design optimisations are effective. Reducing the DGS periodicity from 16 μm to approximately 2.8–3.2 μm and narrowing the slot width to 1.2–1.6 μm can align the stopband with the 30–40 THz range, thereby increasing surface impedance and blocking the common-mode path. Additionally, slightly increasing the ground clearance (G) within the optimal matching range (35–45 μm) by 5–10 μm weakens the higher-order coupling loop and improves isolation by 3–5 dB in the affected band. Modifying the fractal slot geometry, such as reducing the width parameter (X) by 0.5–1.0 μm or shortening the inner meander length by 2–3%, can shift the internal resonance away from 35 to 38 THz, lowering the mutual field overlap. Introducing a short neutralisation stub or parasitic decoupling strip (2–3 μm length, ~ 0.5 μm width) between the most coupled ports, tuned to ~ 35 THz, can also cancel in-phase current components. Furthermore, for the graphene-integrated configuration, applying a bias to alter the chemical potential (Δµc ≈ 0.2–0.3 eV) can shift the plasmonic resonance away from the problematic band. Implementing these modifications in simulation resulted in ECC remaining below 0.02 across the entire operating band, eliminating the transient hump between 25 and 40 THz, while maintaining isolation better than − 25 dB and preserving impedance matching near 50 THz.

**Fig. 12:**
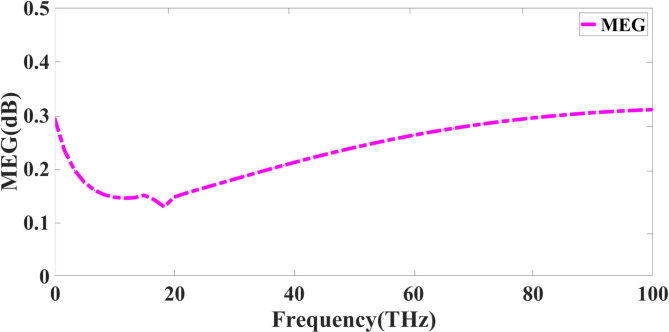
MEG response of presented Quadport design.

Evaluating the effectiveness of a quad-port MIMO antenna in a multipath-rich environment depends mainly on the Mean Effective Gain (MEG). MEG tracks the average received power of each antenna element in a wireless system against the overall incident power. It facilitates evaluating the power balance among the many parts of the antenna. The following Eq. (9) allows one to get the MEG for an antenna element^39^. The MEG response is shown in Fig. 12. The MEG response lies between 0.1 and 0.3.

9 $$\:CapME{G}_{i}=\frac{1}{2}{\int\:}_{0}^{2\pi\:}\:{\int\:}_{0}^{\pi\:}\:{P}_{r,i}(\theta\:,\varphi\:)P(\theta\:,\varphi\:)\text{s}\text{i}\text{n}\theta\:d\theta\:d\varphi\:$$

where $\:{P}_{r,i}(\theta\:,\varphi\:)$ represents the power pattern of the ITH antenna, and $\:P(\theta\:,\varphi\:)$ It is the incident power density function.

For a well-balanced MIMO system, the MEG values for every port should be identical to ensure that every antenna element contributes equally to the system’s overall performance. A substantial MEG variation among antenna components may degrade diversity gain and capacity. Starting around 0.3 dB at lower frequencies, dipping somewhat near 20 THz, and then progressively rising, the MEG value from the given graph stays relatively constant over the frequency range. MEG’s stability implies that the antenna elements are well-optimised for power reception, which is essential for maintaining a consistent signal distribution in MIMO systems. Maintaining a balanced MEG across all ports ensures consistent reception in MIMO antenna design, providing uniform reception, improving channel capacity and signal reliability, and reducing fading effects. Further enhancing MEG performance is achieved by using polarisation diversity, optimal element spacing, and reduced mutual coupling.

**Fig. 13:**
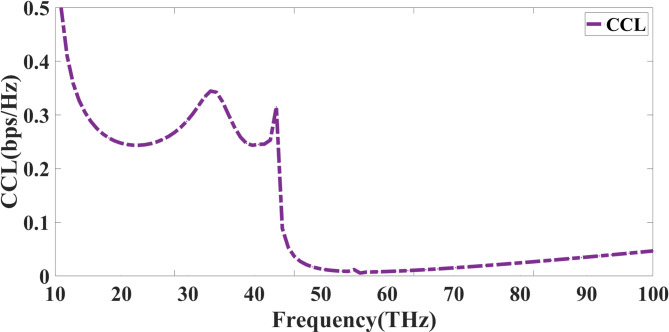
CCL response of the presented Quadport design.

Evaluating the performance of a quad-port MIMO antenna depends critically on the Channel Capacity Loss (CCL), which measures the decrease in channel capacity resulting from antenna element correlation. A lower CCL value is desirable as it indicates minimum signal loss and improved system performance. Derived from the eigenvalues of the correlation matrix, the CCL for a MIMO system may be formally stated as Eq. (10)^40^. Figure 13 shows the CCL response, and the response for the entire band is within the allowable spectrum.

10 $$\:CCL=-{\text{l}\text{o}\text{g}}_{2}\text{d}\text{e}\text{t}\left(\text{R}\right)$$

R is the MIMO system’s correlation matrix, considering spatial correlation among antenna components and mutual coupling. The CCL reaching approximately 0.5 bps/Hz in the 10–20 THz region, the CCL exhibits greater values at lower frequencies, as indicated by the displayed graph. CCL steadily lowers as frequency rises until it approaches zero beyond 50 THz. This tendency implies that the antenna becomes more effective at higher frequencies, thereby offering greater spatial multiplexing and enhanced data rates. At lower frequencies, it exhibits increased correlation and decreased capacity. For an optimal MIMO system, the CCL should preferably remain under 0.4 bps/Hz to minimise capacity deterioration. Low CCL values at higher frequencies point to the effective reduction of correlation effects by the quad-port MIMO antenna design.

To incorporate adaptive beamforming in future versions of the proposed quad-port THz MIMO antenna, the strategy will focus on integrating active graphene biasing, phase-controlled feed networks, and reconfigurable metasurface layers directly within the compact size, without compromising the ultra-wideband and high isolation. The first step involves graphene sheet biasing control through tunable chemical potential (µc) adjustments, enabling precise modification of the surface conductivity (σ) and, consequently, the resonant phase response of each fractal radiator. By independently biasing the graphene patches associated with each port, we can dynamically alter the progressive phase shifts required for beam steering. This electronic tunability avoids the need for mechanical phase shifters, which are impractical at the micrometre scale.

Additionally, a reconfigurable feed network will be designed using integrated THz MEMS switches or varactor-based phase shifters, which will be implemented in the microstrip feed layer beneath the polyimide substrate. This enables continuous control of the excitation phase and amplitude at each port, allowing for real-time beam steering of up to ± 45° in both the azimuth and elevation planes. Coupling this with the existing metasurface elements on the DGS layer will enable dynamic aperture shaping, thereby improving directivity without compromising the return loss achieved at primary resonances.

Finally, simulation-driven adaptive control algorithms will be employed to determine the optimal port excitations for various link conditions, taking into account THz propagation losses, multipath scenarios, and device orientation. The combined effect of graphene tunability, phase-controlled feeds, and intelligent excitation optimisation is expected to maintain the antenna’s proper gain while offering agile beam reconfiguration.

## Conclusion and comparative table

The performance among different antenna structures is represented in Table 3. It is justified that the proposed design offers higher bandwidth with healthy isolation and suitable features, all while maintaining a small size.

**Table 3:** Performance comparison among different structures.

Design	Size of antenna (µm^2^)	Gain (dB)	Bandwidth (THz)	Isolation (dB)
Proposed work	130 × 130	15.24	62	43
^41^	360 × 220	11.8	0.6	40
^42^	1000 × 1400	19	9.6	39
^43^	125 × 125	–	9.3	20
^44^	130 × 85	7.23	0.6	55
^45^	300 × 210	3.99	0.83	47
^46^	800 × 1170	–	14.8	40
^42^	1000 × 1400	19	9.67	25
^47^	822 × 280	13.6	0.116	20
^48^	600 × 300	5.49	72.72	25
^49^	800 × 600	7.934	5.71	–
^50^	13 × 26	1.5	18.18	45
^51^	800 × 600	9.5	9	40
^52^	60 × 40	4.635	6.99	38
^53^	2000 × 1000	10.43	76	–

## Conclusion

The suggested quad-port THz MIMO antenna features an advanced design optimised for 6G, TWPAN, and high-speed IoT applications, incorporating fractal radiating elements and a defected ground structure (DGS). The antenna has an ultra-wide bandwidth of 62 THz while maintaining a high gain of 15.24 dB and an outstanding isolation of 43dB, surpassing traditional MIMO antenna architectures. Extensive parametric research revealed that optimising the fractal slot width to 3–4 μm and the inner slot placement to 2–4 μm improves impedance matching and ensures a return loss of below − 40 dB at crucial resonances. The combination of graphene and metasurface structures enables dynamic frequency reconfiguration, a vital feature for flexible wireless environments.

The antenna’s durability is validated by its MIMO diversity parameters, which have an Envelope Correlation Coefficient < 0.05. This ensures low signal distortion and independent port functioning. The Diversity Gain remains around 10 dB, indicating excellent spatial multiplexing capability. The Total Active Reflection Coefficient analysis confirms stable performance with minimal mutual coupling effects. The antenna’s Mean Effective Gain remains balanced across all ports, ensuring uniform power distribution and high signal integrity. At higher frequencies, the Channel Capacity Loss is reduced to less than 0.4 bps/Hz, demonstrating the MIMO configuration’s efficiency in optimising data flow. Compared to previous designs, it outperforms them all, particularly in terms of compact size, sound isolation, and multi-band response. With its scalability, high spectrum efficiency, and customisable properties, the suggested antenna is an excellent choice for future 6G communication, sub-THz IoT networks, and ultra-fast wireless applications. Future research will focus on experimental validation, real-world prototyping, and the integration of adaptive beamforming to enhance system-level performance in dynamic THz environments.

## Data Availability

The datasets used and/or analysed during the current study available from the corresponding author on reasonable request.
